# Holographic microscopy and microfluidics platform for measuring wall stress and 3D flow over surfaces textured by micro-pillars

**DOI:** 10.1038/srep28753

**Published:** 2016-06-29

**Authors:** Humberto Bocanegra Evans, Serdar Gorumlu, Burak Aksak, Luciano Castillo, Jian Sheng

**Affiliations:** 1 Department of Mechanical Engineering, Texas Tech University, Lubbock, TX 79409, United States

## Abstract

Understanding how fluid flow interacts with micro-textured surfaces is crucial for a broad range of key biological processes and engineering applications including particle dispersion, pathogenic infections, and drag manipulation by surface topology. We use high-speed digital holographic microscopy (DHM) in combination with a correlation based de-noising algorithm to overcome the optical interference generated by surface roughness and to capture a large number of 3D particle trajectories in a microfluidic channel with one surface patterned with micropillars. It allows us to obtain a 3D ensembled velocity field with an uncertainty of 0.06% and 2D wall shear stress distribution at the resolution of ~65 *μPa*. Contrary to laminar flow in most microfluidics, we find that the flow is three-dimensional and complex for the textured microchannel. While the micropillars affect the velocity flow field locally, their presence is felt globally in terms of wall shear stresses at the channel walls. These findings imply that micro-scale mixing and wall stress sensing/manipulation can be achieved through hydro-dynamically smooth but topologically rough micropillars.

It has been widely shown that micro/nano hierarchical structures are vital to functions of biological organs and engineering devices, such as adhesion by gecko feet^1^,^2^,^3^; drag reduction by shark denticles^4^,^5^,^6^; self-cleaning of lotus leafs^7^,^8^,^9^,^10^ and rose petals^11^; and antifouling of textured surfaces^12^. While prior efforts have focused on the development of microfabrication techniques and the understanding of their wetting and adhesion properties, the complex dynamic interactions between flows and these structures have attracted increasing interests due to its importance in understanding the key processes related to human physiology and diseases, e.g. respiratory mucus clearance^13^, gamete transport in the oviduct^14^, right-left symmetry in the embryonic node and cerebrospinal fluid circulation^15^, as well as micro-scale transport/mixing mechanisms employed in microfluidic devices^16^,^17^. Given the scales of the roughness and the flow, transport of mass and mixing is dominated by diffusion. It is well known that the large fluidic features (e.g. serpentine channels, resonant mixing chamber, surface mounted ridges and wells^16^,^17^) can substantially impact processes in microfluidics, e.g. biofilm formation^18^, and micro-scale transport-mixing (chaotic mixing)^17^,^18^,^19^; however, studies on interactions of flow and roughness at more relevant scales, i.e. micro-scale structures in a much larger fluidics, are scarce.

To understand mass and momentum transport over surfaces with micro-scale textures, three-dimensional measurements of fluid and structure motions must be conducted with high spatiotemporal resolutions. Many non-intrusive velocimetry methods have been developed to probe microscale flows. They include micro Particle Image Velocimetry (*μ*PIV)^20^,^21^, scanning confocal microscopy^22^, light microscopy, epi-fluorescent microscopy, and optical coherent tomography. Since an optical microscope only allows flow tracers to be imaged within its depth of field (DOF), it severely limits the resolvability of flow shear in the direction normal to the imaging plane, also known in the *μ*PIV community as the Depth of Correlation (DOC). Detailed explanations are given by Kloosterman *et al.*^23^ and Adrian and Westerweel^24^. Several *μ*PIV methods have been developed to circumvent the issue by encoding the tracer images with depth information^15^,^25^,^26^, but the systems are often complex and difficult to use. Stereoscopic and tomographic *μ*PIV have also been developed^27^ to extract depth information of tracers from multiple projections, however, these systems need at least two cameras and are susceptible to misalignment errors with limited improvement in extending DOF. In addition to the abovementioned imaging challenges, measuring flow in the region very close to a surface (on the order of 1 *μm*) by imaging displacement of flow tracers remains a great challenge to date due to low scattering efficiency of tracer particles and additional noise generated by the refractive index mismatch at the fluid-solid interface. Methods, such as utilizing fluorescent particles as tracers^28^, or matching refractive index of fluids to surfaces^29^,^30^, have been developed to improve particle scattering to background interferences, but remain ineffective to mitigate DOC problem. Zettner and Yoda^31^ applied Total Internal Reflection (TIRF) microscopy to measure the flow velocity as close as 250 *nm* to the wall. The same method was later implemented by Jin *et al.*^32^ to characterize slip velocity over hydrophilic and hydrophobic surfaces. Although powerful, the 3D far-field hydrodynamic interactions are beyond reach with this method.

In-line Digital Holography Microscopy (DHM)^33^,^34^, an inherently three-dimensional recording technique, paved the way for studying many dynamic phenomena by enabling the recording of series of holograms onto a digital camera, while reconstructing them numerically off-line. DHM^34^ has proven to be versatile and robust in recording and resolving three-dimensional motion of particulates or micro-organisms in a volume^14^,^35^,^36^,^37^; in resolving 3-D flow fields around free swimming micro-organisms or small fish^36^,^38^; and in measuring 3D flow structures in the turbulent boundary layer^39^. The technique has been shown to reach a spatial resolution of 200 *nm* over a large depth^34^. Amid the success in imaging planktons and solid particles in 3D, imaging particle tracers over textured surfaces with micropillars in a 3D volume has proven to be difficult due to the overwhelming interferences generated by the difference in index of refraction between surface roughness and nearby fluids. Talapatra and Katz circumvented this problem by matching refractive index of fluids (43 wt% NaI solution) with that of the wall, and successfully measure 3D turbulent flow structures near millimeter scale surface roughness^29^,^30^. However, the difficulty in handling index matching fluids and its associated high toxicity at high concentration often limit its applications. To understand transport dynamics of particles and/or cells near these biomimetic surfaces, a technique capable of imaging 3D motion of micro-scale particles/cells in original biocompatible aqueous solution in the close proximity of surface roughness must be developed.

In this paper, we present the DHM measurement capable of tracking particles (10^6^ particles/ml) over a surface textured with micropillars, resolving the 3D velocity field over it and subsequently quantifying the 2D distribution of apparent skin friction with an estimated measurement uncertainty of 0.06% centerline velocity and 0.9% wall shear stress at the resolution of ~65 *μPa*. The results of 3D velocity distribution and the impact of the micro-scale surface textures (much smaller than microfluidics characteristics) on velocity and wall shear stress distribution are provided in the following section, followed by conclusions and a brief discussion on measurement results and its implications. The experimental methods and details about microfluidic fabrication are provided in the last section.

## Results

### Measurement of instantaneous 3D velocity field using DHM and microfluidics platform

**Figure 1:**
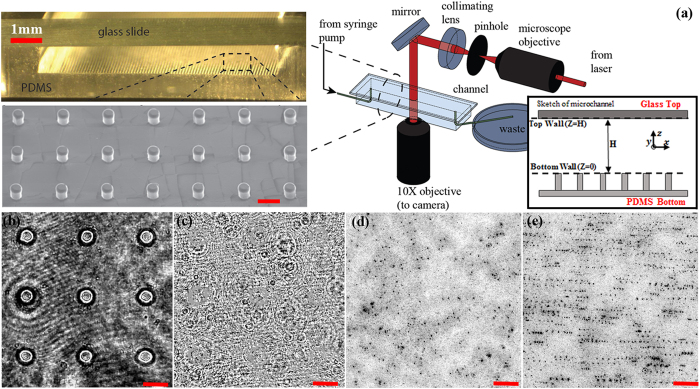
Digital Holographic Microscopy (DHM) and Microfluidics with textured surfaces. (**a**) Schematics of DHM and microfluidic channel. Inset: (Left-top) Micrograph of a cut-away of microfluidic channel showing the glass substrate and PDMS wall. (Left-bottom) SEM micrograph of a PDMS micropillar surface. The lines observed are the fractures developed in a 5-*μm* thick aluminum layer coated over the PDMS for SEM imaging only. The aluminum coating is absent during the experiment. (Right-bottom) Definition of microfluidic walls and coordinate system. (**b**) Original hologram including the interferences of micropillars. (**c**) Particle hologram with micropillar interference removed by correlation-based de-noising algorithm^40^. (**d**) Superposition of particle images reconstructed from the hologram (**b**) at a 2-*μm* interval over the entire depth of 1,420 μm. (**e**) Superimposed in-focus particle reconstructions over seven consecutive time steps showing the particle streaks. Scale: 50 *μm* or otherwise specified.

The experimental setup (Fig. 1a) consists of a DHM^40^ and a microfluidic channel with the surface texture. Details are provided in Section Methods as well as illustrated in Fig. 1a and Table 1. Several flow conditions are investigated (Table 2). Focusing primarily on methodology, in this paper we only present results from the second case (highlighted in Table 2). For clarity, we refer to the “apparent” top and bottom walls as surface-parallel planes indicated in the inset of Fig. 1a. To differentiate, we refer to the physical walls as substrates. Note that the apparent bottom wall (*z* = 0) is the ‘canopy’ plane passing through the tips of the pillars. Hence, the apparent bottom wall is composed of the solid patches at the PDMS pillar tip (subscript “*mp*”) and the fluid interfaces in between (subscript “*fd*”), whereas the apparent top wall (*z* = *H*) is the continuous smooth glass. The flow and wall shear stress distributions are measured and approximated at these two apparent walls. For brevity, we will omit “apparent” hereinafter. The coordinates are defined as streamwise (*x*), spanwise (*y*) and wall-normal (*z*) with corresponding velocity components as *u*, *v*, and *w,* respectively.

Figure 1b shows a small portion of a recorded sample hologram with the imaging plane of DHM located 60 *μm* below the bottom wall (*z* = −60 *μm*). Note that the recorded holograms are the out-of-focus interference patterns of particles and pillars throughout the entire measurement volume. The interference of the pillars are clearly overwhelming those of tracer particles. This low fringe contrast severely limits our ability to accurately resolve positions and motions of tracer particles and hence hinders the accurate flow measurements near the rough surface. Talapatra and Katz^29^,^30^ have circumvented this problem by matching the index of refraction of working fluids with that of the roughness, such that the boundary between the fluids and roughness disappears. Although effective, these fluids are often difficult to handle, which limits the applicability of the method. In this paper, we applied an algorithm that allows us to effectively separate the interferences of pillars from those of flow tracers directly, and substantially enhance the fringe contrast of tracer particles (see Fig. 1c), allowing accurate measurement of particle position and motion very close to a rough boundary. Based on observations, interferences of pillars are spatially and temporally correlated, which differs greatly from the randomness of the tracer interferences. In comparison to tracer interference, those pillar interferences can be considered as correlated noise. Therefore, a background hologram containing only the interference of pillars and laser instability for each individual hologram can be successfully estimated using the in-house developed correlation based de-noising algorithm, which has been reported in our recent publication^40^ and been applied to successfully image bacteria cells in a 3D volume^14^. Figure 1c shows the “cleaned” hologram to the same portion of the original sample hologram (Fig. 1b). The separation and the enhancement of tracer hologram with the absence of the interferences originated by pillars is clearly evident. This procedure paves the way for accurate measurement of particle positions and displacement in the immediate vicinity of the roughness.

The techniques to measure 3D velocity fields from DHM recordings are extensively documented^33^ and have been successfully applied to measure 3D flows in a turbulent boundary layer over a smooth^39^ and a rough wall^29^. We will only summarize procedures used in the present study: The optical field containing in-focus particle images are first reconstructed numerically using Fresnel Integral^33^ with an interval of 2 *μm* over the entire channel depth of 1.4 *mm*. The in-focus particle images are reconstructed from the same hologram (Fig. 1c) and collapsed over the depth to show all particles within the volume (Fig. 1d). Figure 1e shows the superimposed particle image over seven consecutive time frames; the motion of flow is clearly elucidated. The detailed particle information, shape and position, is extracted using the 3D segmentation routine^39^. Approximately 3500 particles are identified in each time frame. Reconstructed particles are tracked over time using a PIV-assisted particle tracking algorithm^39^. Although the position and motion of particles within the roughness are clearly reconstructed and captured from the “cleaned” hologram, as evidently shown in the subsection on conditionally sampled 3D flow field later, resolving high resolution velocity measurement within the roughness remains difficult due to low tracer seeding density. At present concentration, only the flow field above the apparent wall can be adequately resolved and will be discussed in this paper.

**Figure 2:**
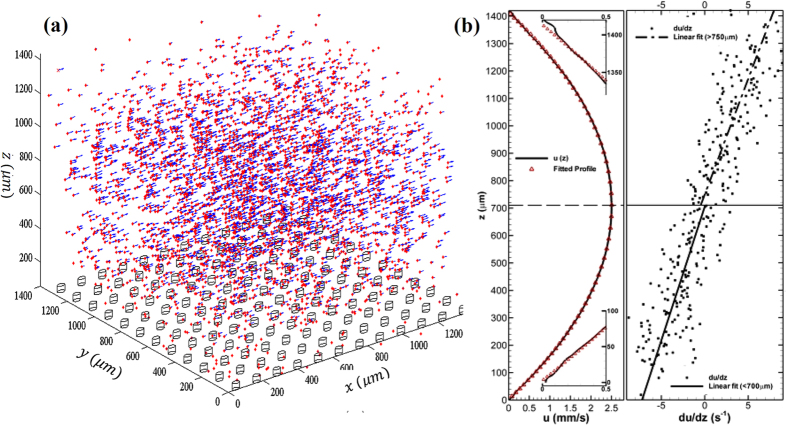
Measurement of an instantaneous and mean velocity fields. (**a**) An instantaneous velocity measurement over a textured bottom surface obtained by Particle Tracking Velocimetry (PTV). The micropillars are shown as cylindrical posts. Vectors are marked by an arrow started by a red dot. (**b**) Mean velocity profile of streamwise component and velocity gradient distribution along the depth direction. (Left) 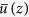 averaged over a 1 *μm* thick surface parallel layer with entire lateral dimensions. Solid line: measurements, Symbol: Least square fit of a parabolic profile. Insets: Close-ups of 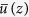 at both surfaces. Except for close proximity to the wall (<30 *μm*), good agreement between measurements and the fitted profile has been found. (Right) distribution of the flow shear, 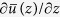. Symbols: estimations by differencing the velocity profile shown in (Left) by Central Differencing Scheme (CDS); Solid Line: Linear regression of flow shear in the lower half of the channel; Dash Line: Linear regression in the upper half of the channel.

A sample measurement of 3D instantaneous velocity (>3500 vectors in a volume of 1.4 × 1.4 × 1.4 *mm*^3^) is shown in Fig. 2a. The magnitude of instantaneous velocity field shows anecdotally a Poiseuille-like flow pattern common to microfluidics. We estimate that the particle positioning accuracy of 0.1 pixel (or 0.14 *μm*) in both lateral (*x-y*) directions where a sub-pixel resolution is achieved by fitting a parabolic distribution over a 5 × 5 pixel in-focus image around the centroid calculated by the weighted mean of 3D particle scattering (details refers to ref. 34) and 0.5 tracer diameter (or 1 *μm*) in the depth (*z*) direction. The depth position uncertainty of one particle diameter using DHM has been well documented and substantiated^29^,^40^,^41^. We estimate that the measurement uncertainty of instantaneous velocity is at ±14 *μm*/*s* (0.1 pixel displacement uncertainty) in the lateral directions (*x* and *y* axis) and ±20 *μm*/*s* in the depth direction (*z* axis), which translates to 0.6% and 0.8% of the centerline velocity in the in-plane and out-of-plane directions, respectively. The uncertainty (standard error) is given by *σ*/(*N*)^1/2^, where *σ* is the standard deviation of the *N* vectors found within a 2 *µm* slice. Further analysis on uncertainty normalized by the local streamwise velocity, *u*(*z*), reveals that the local relative error peaks at both walls with a value of 1.5%.

### Measurement of 3D flow field and wall shear stress distribution

The ensemble averaged velocity field is computed over 1000 instantaneous velocity fields, totaling over 3.5 × 10^6^ vectors. The 3D mean velocity fields are determined using two different methods and compared with each other. The first method uses a simple volume average over a rectangular volume centered at the point, *x*_*i*_, as


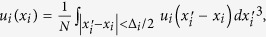


where *N* is the number of vector within the averaging volume, Δ_*i*_ is the *i*-th dimension of the volume, and the index, *i* = 1, 2, and 3. We select an averaging volume of 60 *μm* × 60 *μm* × 2 *μm* that contains a minimum 200 velocity vectors. The measurement uncertainties of ensemble velocity (i.e. 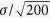) is 1 *μm*/*s* (or 0.04%) for the lateral components and ±1.4 *μm*/*s* (or 0.06%) for the depth component. Although volume averaging is a simple method, and well known for its inability to resolve accurate the flow near the wall^29^, large-scale flow structures (e.g. mean profile far away from the wall) are adequately resolved, and thus provides the baseline for comparison.

However, to elucidate the near surface flow at micron scales and resolve the 3D flow near the roughness, a more accurate method based on an interpolation scheme using Taylor expansion^39^ has been applied (Fig. 2). The accuracy and the unique capability of this method in comparison to other interpolation schemes have been investigated and reported by Talapatra and Katz^30^. In the current paper, each interpolated velocity vector near the surface is computed from at least 500 data points, the uncertainties are estimated as 0.62 *μm*/s (or 0.02% of *U*_*c*_) and 0.83 *μm*/s (or 0.04%) in the in-plane and out-of-plane directions, respectively.

**Figure 3:**
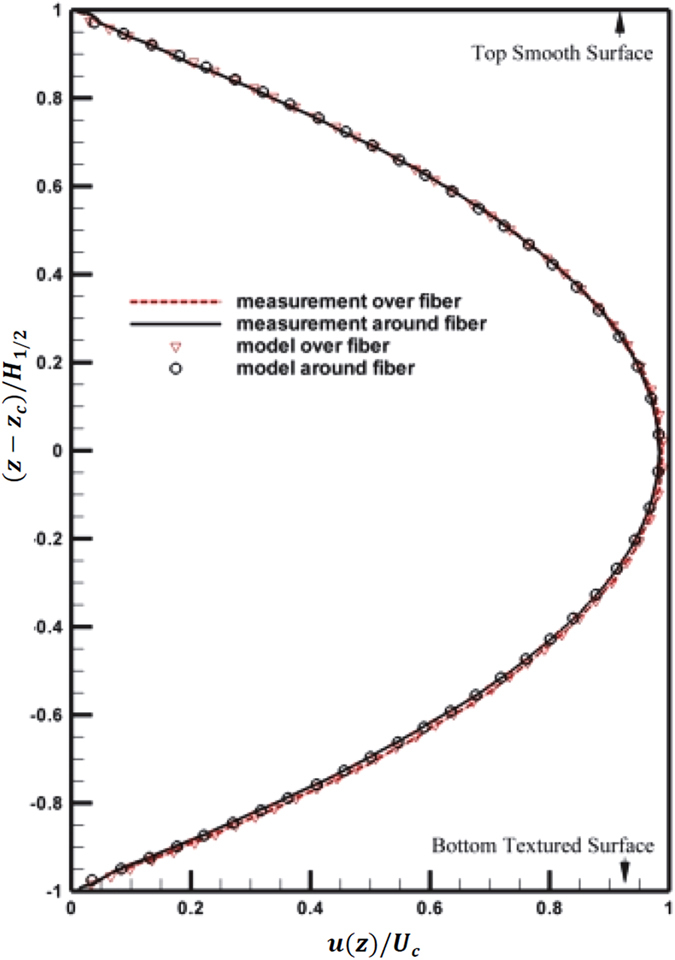
Mean streamwise velocity profiles and least square fit (LSF) of local Poiseille flow models. Normalized velocity profiles: (Solid line) – over the solid surface at the micropillar tip, (Dashed line) – over the fluidic interface separates nearby micropillars. Symbols: LSF model (Eqn. 3).

Accurately quantifying wall shear stresses over a textured surface such as the one in the study has vital implications in assessing the dynamic properties of a functionalized surface, such as wetting, friction and adhesion over them. However, due to the presence of high flow shear and overwhelming Brownian motions by tracer particles in comparison to the flow advection near the wall, conventional methods that employ a direct estimate of the tangent of the velocity profile at the wall are expected to be inaccurate. Note that the mean velocity profile shown in Fig. 2b, obtained by the volume averaging method, introduces large fluctuations and relative measurement uncertainty near both surfaces. In literature, many methods have been developed to approximate the wall shear stress for the near wall velocity profile, i.e. 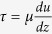, where *μ* is the kinematic viscosity and, 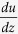 is the flow stress tangent to the wall. Among them, the evaluation of the velocity gradient directly at the wall is most widely used. However, this method mandates high resolution measurement of the velocity profiles near the wall. It has been shown by Talapatra and Katz^29^ that velocity profile obtained by volume averaging methods including those used by PIV and µPIV, yield an inaccurate velocity profile near a strongly sheared wall. Subsequently, they have concluded that PTV analysis yielding an unstructured displacement field of tracer particles results in much more accurate mean velocity distribution near a wall. The same approach has been adopted in the current paper. Although resolution and accuracy are greatly improved using the above-mentioned approach, direct differentiation of velocity profile remains challenging, since the estimation relies solely on those data points close to the wall where large fluctuations and uncertainties are expected. To incorporate more data points in the *z* axis, Sheng *et al.*^30^ have developed a method to measure local instantaneous wall shear stress in a turbulent boundary layer (TBL) taking advantage of the fact that TBL has constant flow shear stresses, *τ*_*x*_, (or linear velocity profile) within a region of 5 wall units away from the wall (or 60 *µm* in ref. 39), also well known as the viscous sublayer. To estimate the instantaneous wall shear stress distribution, a linear fit was performed over the scattered velocity measurements within the viscous sublayer^39^,^42^. The same method has been successfully applied to rough wall turbulent boundary layer flow and obtained highly accurate shear stress distributions at roughness canopy^29^,^30^. The success of these studies highlights the importance in incorporating near wall flow characteristics in the estimation of local wall shear stresses. Following the same principle, we have developed a method based on finding the linear regression of flow shear along the depth direction and computing its value at the “apparent” wall location. For instance, to compute a streamwise wall shear stress, *τ*_*x*_(*x*, *y*), a vertical mean velocity profile, 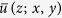, will be computed locally as follows,


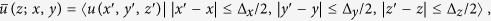


where 〈⋅〉 denotes volume average, and Δ_*x*,*y*,*z*_ are the dimension of the averaging volume in *x*, *y* and *z* direction, respectively. This method is based on our observations that local vertical mean velocity profiles right above the different bottom textures remain parabolic (Fig. 2b) and the flow shear stress, 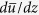, varies linearly in the *z* axis, but differ distinguishably in the slope of flow shear stress (right panel in Figs 2 and 3). This suggests that 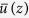, even far away from the surface, contains local texture information, hence can be used to approximate the stress distributions.

Figure 2b depicts the mean streamwise velocity profile obtained over a series of averaging volume of 1.4 *mm* × 1.4 *mm* × 1 *μm* along the *z* axis, the latter being the depth, and the applied quadratic fit (symbol). The streamwise mean velocity profile is parabolic except for those regions in the extreme proximity (~40 *μm*) to both walls. The wall normal velocity gradient, 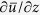, is evaluated numerically from the profile, 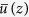, at various *z* locations (right panels in Fig. 2b) using the central finite differencing scheme. The linear regression is performed over 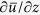 distribution and the wall shear is evaluated as the boundary value of the regression. To accommodate the different boundary conditions present in the top and bottom wall of the channel, we perform the regression over the upper and lower half of the channel separately. The difference in flow shear distribution in the upper (dash-dot line) and lower (solid line) half of the channel is clearly shown in Fig. 2b, and consequently results in different wall shear stress between the upper and the lower walls. It can be demonstrated that the 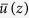 (Fig. 2b) contains texture information but remains Poiseuille flow, and hence can be used to estimate wall shear stress in microfluidics. This also suggests that flow shear, including wall shear stress, must vary linearly along the *z* axis, which provides us a much robust means to estimate wall shear stress. In comparison to conventional methods that approximate the stress by directly evaluating the velocity gradient at the wall, the proposed method is less sensitive to errors in determining wall positions and near wall velocity, and is expected to reach more accurate and robust estimation. This observation of Poiseuille-like 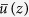 in general but differing over textures, will be further discussed in the following section. The range of the linear regression on 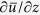, is chosen by varying the range from 50 *µm* to the half of the channel height from the wall. We found minor differences in wall shear stress. For simplicity, we use the entire half channel for linear regression calculation. To estimate the local wall shear stresses, *τ*_*x*_ and *τ*_*y*_, the above mentioned method is applied to the local mean velocity, 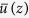, which is calculated over a local averaging volume sufficiently small to yield statistically robust local mean velocity. To estimate the uncertainty of 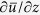, we use the twice of the maximum uncertainty of the velocity, approximated by Taylor expansion method (i.e. 2 × 0.83*μm*/*s*) and divided by the minimum depth that yields a stable linear regression (i.e. 50 *μm*). It is estimated at 0.033 *s*^−1^. To be more conservative, the resolution of flow shear is set as twice of flow shear uncertainty at 0.066 *s*^−1^ (or 0.9% of wall shear) and subsequently the resolution of 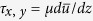 at ~65 *μPa*. Note that this estimation is very conservative and highly depends on the uncertainty of the velocity measurement.

### Mean flow velocity in microfluidics with smooth and textured surfaces

To study the effects of microscale textures on near wall flow, especially the mean statistics, we first examine mean streamwise velocity distributions along the *z*-axis (Fig. 2b). The mean streamwise velocity, 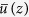, averaged over both lateral (*x* and *y*) directions, assumes a clear parabolic form that suggests a Poiseuille-like mean flow within the micro-channel. Since the characteristic length scales of flow, e.g. the height (*H*) and length (*L*) of the channel, are much larger than those of pillars, i.e. *H*/*h*_*mp*_ = 36, *L*/*d*_*mp*_ = 1125 and *W*/*d*_*mp*_ = 250, dimensional analysis can show that the mean flow within the current channel is driven by mean streamwise pressure gradient, 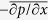, where the overbar indicates the spatial average in *x* and *y* directions. The governing equation for mean flow parameters reduces to 1D Hagen-Poiseuille equation,


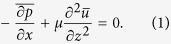


Hence, the mean flow must satisfy Hagen-Poiseuille solutions and assume a parabolic profile along the *z* direction, while the inhomogeneous bottom wall composed of patches of solid pillar top surfaces and fluid interfaces in between can be treated as a superposition of the corresponding boundary conditions. For instance, the boundary condition over the pillar top is treated as the no-slip boundary condition, *u*_*mp*_ = 0, and that over the fluid interface as the shear stress boundary condition, *u*_*fd*_ ≠ 0 and ∂*u*_*fd*_/∂*z* = *τ*_*fd*_/ *μ*.

Based on observations (Fig. 2b), one can further speculate that owing to the periodic distribution of pillars, the presence of pillars must be felt throughout the entire channel. It can strongly affect the flow characteristics in the *z*-direction but weak dependency on both lateral directions, i.e. 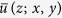 at a given location, (*x*, *y*), which can only be modified by local surface conditions (e.g. solid surface or fluid interface). To validate this hypothesis, we compute the ensemble averaged velocity profiles, 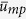 and 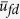. 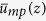 is the velocity profile averaged within the fluid column right over the tip of pillars,


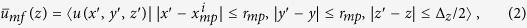


where 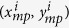 is the center of the *i*-th circular cross-sectioned pillar with a radius of *r*_*mp*_; and 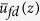 is obtained over the column right above those areas excluding the tip of pillars. The boundary conditions for 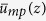 and 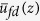 are different. At the bottom surface, fluids right above the pillar tip encounter solid surface, hence the no-slip boundary condition must be applied to 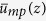; whereas the stress boundary is imposed upon 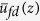. The profiles, 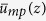 and 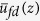 are shown as the dashed and solid line respectively in Fig. 3. Note that both profiles coincide well in the upper half and deviate substantially from each other in the lower half of the channel, except for the region (0.95 − 1*H*_1/2_, where *H*_1/2_ is the half channel height). Notice that a 5% of velocity increase between 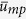 and 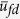. It can be accentuated that the upper-half profiles collapse when the nearest boundary condition is homogeneous, and the lower-half profiles differ substantially over heterogeneous boundaries. Furthermore, such a difference in boundary conditions appears to affect only the mean flow in the *z*-direction (the only non-homogeneous flow direction).

An analytical model has been developed to validate the abovementioned hypothesis: local surface conditions only affect the local velocity profile. Owing to the high aspect ratio (*W*/*H* = 7.14) and small normalized pillar height (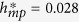), the Navier-Stokes equation can be simplified to the Hagen-Poiseuille equation (Eqn. 1). The top surface is prescribed as no-slip boundary condition (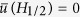), whereas the bottom rough surface as shear stress distribution *τ*_*B*_ with periodic patches of *τ*_*mp*_ and *τ*_*fd*_. The velocity profile, 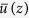, can be solved analytically to yield the following profile:


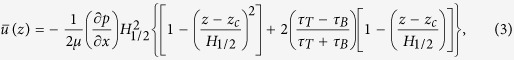


where *τ* is surface shear stress, *z*_*c*_ is the depth location of the channel center line (i.e. *z*_*c*_ = *H*_1/2_), subscript *T* and *B* denote the top and bottom surface. It must be stressed that Eqn. 3 is one-dimensional and only applicable for the bulk flow. A model based on Hele-Shaw approximation is needed to model the flow below the canopy. It can be shown that the pressure force over unit section of microfluidics is balanced by frictional surface force, 
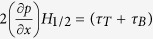
. In Eqn. 3, the first term is the quadratic solution to Poiseuille equation with two no-slip surfaces, and the second is the linear solution necessary to match two shear stresses at the boundaries. We fitted the model over 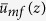 and 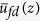, and estimated the shear parameter,


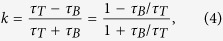


by applying the least square fit. The ratio of shear stress, *τ*_*B*_/*τ*_*T*_, at the top and bottom surfaces over pillar and flow interface are measured as 0.9996 and 0.9951, respectively. The corresponding normalized velocity profiles over different bottom patches are evaluated using estimated *k* parameter and plotted in Fig. 3. The model and measurements agree very well throughout the entire depth. Note additionally that *τ*_*B*_/*τ*_*T*_ over pillar is larger than that over fluids interface, which agrees with the fact that the friction over a solid is larger than that over fluid interface. It should be pointed out that the shear stress at the bottom rough surface is lower than that at the top glass surface.

### Conditionally sampled 3D flow structures and 2D shear stress distributions over a single pillar

**Figure 4:**
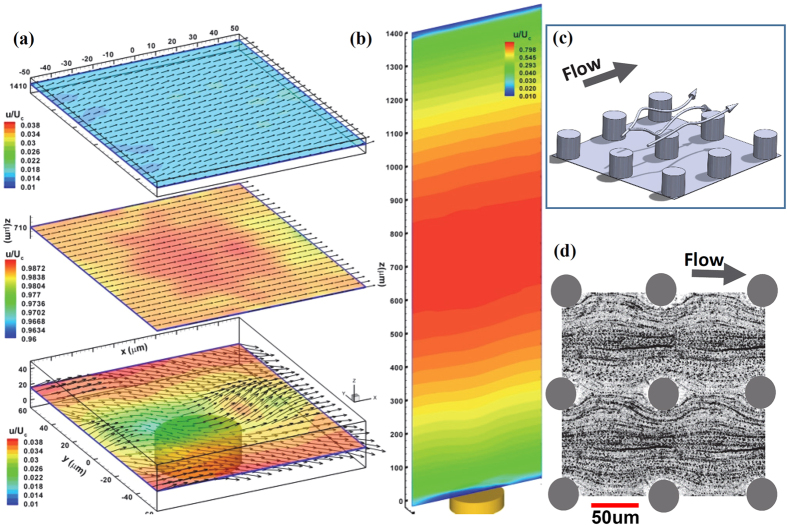
3D ensemble flow right above and beneath a single micropillar in microfluidics. (**a**) The *x-y* slices of a 3D velocity field, *u*/*U*_*c*_, where *U*_*c*_ is the centerline streamwise velocity, superimposed over the contours of velocity magnitude at z = 10 μm (10 μm to the bottom rough wall), 710 μm (center channel), and 1410 *μm* (10 *μm* to the top smooth wall). (**b**) The streamwise velocity magnitude at the *x-z* plane passing through the center of the pillar (*y*_*r*_ = 0). (**c**) A conceptual elucidation of complex 3D flow structures (marked by streamlines) around a single micropillar. (**d**) Tracer streaks show the Stokes like flow beneath the pillar canopy. The image is obtained by superimposing the collapsed reconstructed particle images between micropillars over the entire time sequences at an interval of every 5 frames. Only image among a 3 × 3 micropillars is shown for clarity.

To further understand the effect of the pillar on hydrodynamics, we performed conditional sampling to generate the ensemble flow field around a single pillar. The PTV measurements were re-mapped into a repeating sample volume centered at a single pillar using the procedure, 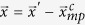, where 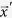 is the position of the velocity measurement, 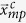 is the center position of the closest pillar and 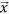 is the remapped location. The volume has dimensions 120 *μm* × 120 *μm* × 1400 *μm* (the latter being the height). The ensemble velocity field on regular Cartesian grids was interpolated from scattered measurements using Taylor expansion^39^. Three *x-y* planes near the bottom (*z* = 10 *μm*), middle (710 *μm*), and top (1410 *μm*) of the channel are shown in Fig. 4a and the streamwise velocity magnitude in the *x-z* plane (Fig. 4b) centered at the pillar (shown as the golden post). The 3D velocity vector maps are superimposed with the contour maps of the streamwise velocity component.

The flow in the vicinity of the pillar is highly three-dimensional (Fig. 4a). Near the bottom surface, in streamwise direction, high speed flow is observed between pillars, and a low speed flow right over it. In the high speed streak regions (high pressure zones), the flow accelerates before the pillar and sweeps into the cavity below the “canopy” as passing through the pillar. In the low speed streak region (low pressure zone), a series of acceleration and deceleration events are evident. Before encountering the pillar, the flow slows down substantially and sweeps into the pillar surface (high pressure); as it passes the pillar, the flow “lifts” away from the canopy plane right behind the pillar (low pressure). This ensemble flow field will repeat itself and generates a periodic wavy flow near the roughness, and subsequently corresponding pressure variations in both streamwise and spanwise directions with length scales similar to the pillar spacing. This unexpected turbulence-like flow features at the leading and trailing edge of the pillar can be in fact explained by the continuity of the incompressible flow. At the *x-y* plane located at the center of a micro-pillar, 2D continuity can be assumed, ∂*u*/∂*x* + ∂*w*/∂*z* = 0. At the leading edge, since the microchannel contracts, the flow must accelerate (i.e. ∂*u*/∂*x* > 0), which leads to ∂*w*/∂*z* < 0, and hence a negative *w* component corresponding to a “sweep” event; whereas at the trailing edge, the channel expands, which leads to deceleration (i.e. ∂*u*/∂*x* < 0), and consequently ∂*w*/∂*z* > 0, corresponding to an ejection event. As one moves away from the bottom, 3D flow gradually returns to 2D laminar pattern. One should note that the imprint of the pillar is persistent in the lower half of the channel. Although the signature in the stream-wise velocity field is weak in the middle of the channel, the high-speed flow region with a rough imprint of the pillar is clearly observed (center plane). However, the signature in the flow diminishes as it approaches the upper wall. The variation of the velocity field in the depth direction is clearly elucidated in Fig. 4. Note that the depression of the contours is clearly observable in the lower half of the channel, while uniform contour distributions are present in the upper half of the channel. Figure 4c shows the conceptual 3D flow over a single pillar within a microfluidic channel. Moreover, Fig. 4d depicts the velocity of the particle streaks in the gaps beneath the canopy integrated over the entire height of the pillar. A Stokes-like flow pattern is clearly observed. This observation has strong implications in fundamental principles of micro-scale mass and momentum transport over complex surfaces, hydrodynamic properties of textured surfaces, and, most importantly, subtle interplay between surface micro-structures and flow characteristics: e.g. pressure and momentum.

**Figure 5:**
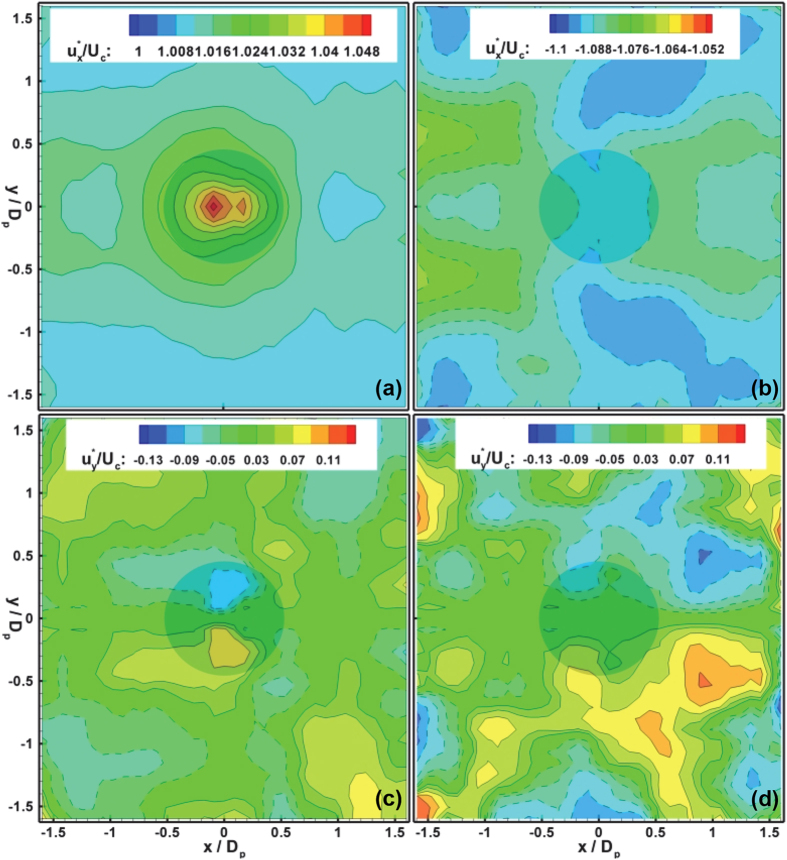
2D Distributions of frictional velocity, 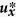 and 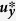, normalized by the centerline velocity, *U*_*c*_. The distributions of 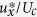 at (**a**) the bottom rough and (**b**) the top smooth surfaces. The distributions of 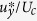 at (**c**) the bottom rough and (**d**) the top smooth surfaces. The position and dimension of the micropillar is shown as a shaded circular area.

Equipped with direct shear estimation method, the 2D shear stress fields (*τ*_*x*_, *τ*_*y*_) are computed over both the bottom and top walls. Each measurement is computed from the mean velocity profiles constructed over an averaging volume of 30 *μm* × 30 *μm* × 1 *μm*. The mean wall shear stress is then estimated at 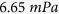 at the rough bottom and 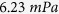 at the smooth top wall, respectively. Following the convention of studies on skin friction and transport over surfaces as well as laying the foundation to study the scaling laws of transport in textured microfluidics, we present the wall shear stress distribution, (*τ*_*x*_, *τ*_*y*_), in the form of friction velocity or shear velocity, u^*^, normalized by the characteristic flow velocity. This non-dimensionalized quantity compares shear-related motion with characteristic flow motions. The friction velocities are computed as 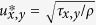, where the subscript, *x* and *y*, indicates streamwise and spanwise, respectively, and normalized by the centerline velocity, *U*_*c*_. Although the *x-y* distributions of normalized frictional velocity, 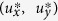, show reflection symmetry, there is clearly a lack of streamwise symmetry mandated by a flow in the low Re regime (Fig. 5). It is clear that 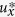 increases rapidly as it approaches the center of the pillar but recovers slowly. Recall that this local friction distributions matches very well with the near wall flow shown in Fig. 4, i.e. sweeping flow correlates with high friction region and ejection flow with the low friction region.

In addition, the high speed flow regions on sides of the pillar show clear low viscous shear stresses in comparison to that over the solid tip of the pillar. It can be observed that although the velocity field at the top shows no imprints of the bottom roughness, the frictional velocity shows a clear texture reminiscent of the surface roughness, i.e. correlated distributions with the streamwise and lateral spacing similar to that of the roughness. Since the local surface friction is directly balanced by the lateral pressure gradients in low Re flow, it clearly shows that the surface roughness affects the momentum only locally, but the viscous stresses affect the entire depth. A point of interest arises that 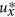
(~1.075*U*_*c*_) and consequently *τ*_*x*_ at the top surface is surprisingly larger than that 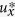
(~1.03*U*_*c*_) at the bottom surface (~4% reduction in viscous stress), whilst 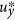, hence *τ*_*y*_ are comparable at both surfaces. This is expected given that a solid boundary is imposed by the cylinder and a slip boundary condition is generated by the fluid within the roughness layer. Note that the aforementioned friction is only computed over the apparent bottom wall. If the total skin friction is a concern, the contributions by those surfaces below the canopy must be counted and are expected to exceed the viscous stress at the top smooth wall. The present results have unparalleled implications in skin friction modelling, prediction and management techniques in different flow regimes, as well as in understanding new sorption mechanisms of particles, micro-organisms and cells over textured surfaces under various flows.

## Discussion

In the paper, we have presented a approach to simultaneously measure the 3D flow in a microfluidic device and wall shear stresses over both smooth and micro-textured substrates at the micro-channel’s top and bottom surfaces, respectively. A microfabrication method to manufacture the PDMS device and pattern the channel surface concurrently has been developed to produce seamless integration of micro-scale surface functionalization with a microfluidic device. Contrary to conventional fabrication methods of PDMS microfluidics, a polymeric master with negative surface texture is fabricated and then used to mold out the micro-textured microfluidic channel. This method is proven to be simple and robust, and thus allows us to create uniform textures over large scales of the microfluidic wall. This capability uniquely permits us to explore the effects of pillars on near-surface hydrodynamics as well as the particle-roughness interactions. The 3D flow and subsequently viscous stresses over surfaces are measured directly using DHM. The flow field is obtained by tracking 1.9 *μm* tracer particles in a 3D sample volume. To overcome difficulties in imaging particles in 3D through the interferences of surface roughness, a novel DHM technique capable of numerically removing the interferences of pillars from the particle hologram have been developed. We have shown for the first time that DHM with correlation-based de-noising method^40^ is capable of measuring the particle field over complex boundaries without applying existing index matching techniques. The high speed DHM measurement over a sample volume of 1.4 × 1.4 × 1.4 *mm*^3^ is performed at 200 fps under the centerline velocity of 2.4 mm/s. Over 3000 displacement vectors per recording have been obtained, totaling 3 × 10^6^ vectors for a series of 1000 holograms, with a position accuracy of 0.14 *μm* and ~1 *μm* in the in-plane and out-of-plane directions, respectively, with corresponding instantaneous velocity measurement uncertainties of 14 *μm*/*s* and 20 *μm*/s. The ensemble average of the 3D velocity field with uncertainties of 0.62 *μm*/*s* and 0.83  *μm*/*s* is obtained to generate the calculation of local mean velocity profiles and direct estimation of the shear stress distributions over surfaces within the microfluidics. The measurement resolution of shear stress is estimated at ~65 *μPa*.

Our results show that the flow over a textured surface in a microfluidic device can be highly three-dimensional and complex, even in the case of small roughness (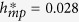). The ensemble flow clearly shows the complexity of the secondary flow structures around a single pillar not observed in microfluidic devices, i.e. low and high speed streaks, sweep and ejection flow events. The impact of roughness on the momentum field is localized and only strongly influences the nearest half of channel, while the pillars affect the pressure field throughout the entire depth. The viscous stress distribution, on the other hand, is the result of the subtle interplay between far-reaching pressure gradient and localized viscous stresses. These observations have implications in basic principles of the skin friction generation over micro-scale roughness (or hydro-dynamically smooth surface), and mass-momentum transport near these surfaces. They may lead to effective fluid transport in a wide-range of flow regimes, as well as new techniques to manipulate mass transport and mixing at micro-scales.

## Methods

The experiments were performed in a straight microchannel, which has one of its horizontal walls textured with polydimethylsiloxane (PDMS) pillars (Left panel in Fig. 1a, dimensions of the channel in Table 1), while the second is a smooth substrate. The channel (Table 1) with functionalized surfaces (top: smooth, bottom: roughness) allows us to conduct comparative investigations of viscous stresses and to uncover 3D flow structures over these surfaces. In this section, we will first describe fabrication method followed by velocimetry methods^14^,^40^.

### Channel fabrication and surface functionalization

To allow hydrodynamic impact of the surface texture on fluid flow, the device and its internal surfaces must be fabricated and functionalized simultaneously. The fabrication method is derived from the widely used PDMS molding over a SU8 master^43^. The channel fabrication process consists of two general steps: (i) The preparation of a soft master mold, and (ii) fabrication of microchannel.

#### Preparation of a soft master mold

A SU8 layer (MicroChem, Inc) of a desirable thickness, i.e. 40 *μm*, which is equivalent to the height of the micropillars, is spin-coated onto a SI wafer. The photoresist is patterned by soft-lithography^1^,^43^,^44^ developed and pillars revealed. A layer of PDMS (1:10 reagent to polymer ratio) with the desirable thickness of 1.4 mm, which is equivalent to the height of the microfluidic channel, *H*, is deposited on the patterned wafer to create the soft master mold (i.e. pillars form cylindrical holes in a master mold). Precise control of the thickness can be achieved by various depositing methods. The mold is cured over night at 80 °C and released from the wafer surface. The PDMS master mold is bonded with a glass substrate using plasma activation (Oxygen plasma at 100sccm, 80 W for 1 min, Technique RIE 8000).

#### Fabrication of textured microchannel

The PDMS master mold is salinized by Trichloro(1H,1H,2H,2H-perfluorooctyl)silane (Sigma Aldrich, USA) in a vacuum chamber overnight as a stress relief agent in order to prevent PDMS bonding to the master mold during channel fabrication. Additional PDMS is deposited over the soft master to form the microchannel. After curing at 80 °C overnight and releasing from the master mold, the PDMS channel with a textured surface is bonded permanently to a glass substrate using oxygen plasma activation (oxygen plasma under 100 sccm, 80 W for 1 min, Technique RIE 8000). The inset in Fig. 1a shows the cut-away of microfluidics. Uniform pillars over the bottom polymer substrate are shown in SEM micrograph (Bottom inset in Fig. 1a). Note that the irregular “patterns” and “lines” observed on the bottom substrate are fractures of a 5-*µm* thick Al layer sputtered for SEM visualization purpose only. During the experiments, this Al layer is absent and the bottom surface at the base of the micropillars is smooth. The geometric parameters are summarized in Table 1.

### 3D flow measurement and data analysis procedure using DHM with background image removal

#### Experimental setup

The setup, as shown in Fig. 1a, consists of a 15 mW CW He-Ne laser (*λ* = 632.5 *nm*), an inverted microscope (Nikon TS-100) and collimating optics to generate a spatially uniform and collimated beam. A series of holograms were recorded with a 1 K × 1 K high speed CMOS camera (IDT-N3, IDT) with a pixel size of 13.68 *μm*. The holograms were imaged at a magnification of 10X with its imaging plane located at the plane 120 *μm* below the bottom wall (small portion of a sample hologram shown in Fig. 1b), resulting in a sample volume of 1.4 *mm* × 1.4 *mm* × 1.4 *mm* and a spatial resolution of 0.7 *μm* and 2 *μm* in the lateral and depth directions, respectively. The achievable imaging resolutions by DHM are well documented in Sheng *et al.*^34^ and Molaei and Sheng^40^. Filtered DI water was seeded with 1.5 *μm* polystyrene particles (Duke Scientific) and injected into the microfluidic channel by a syringe pump (NE-100, New Era Pump System, Inc.) Several flow conditions were investigated (Table 2). Suitable recording rates were selected to maintain the maximum particle displacement not exceeding 20 pixels.

#### Inertia effects of tracer particles in current microfluidics

In microfluidics, the inertia of particles can be amplified due to strong flow shear and close proximity to fluidic walls and causes them to deviate from the flow streamlines. A suite of microfluidic devices, namely inertial microfluidics, has been developed for micro-scale particle separation and sorting applications^45^,^46^. To assess the inertia of particles interacting with flow, we use dimensionless Stokes number (*St* = *τ*_*p*_*U*_*c*_/*H*, where *τ*_*p*_ is the characteristic time of a particle, often using the particle relaxation time, 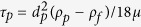 in fluid mechanics) and particle Reynolds number (*Re*_*p*_ = *Re*_*H*_{*d*_*p*_/*H*}^2^, where *Re*_*H*_ the flow Re. Both dimensionless quantities are much less than the unity as *St* ~ *O*(10^−7^) and *Re*_*p*_ ~ *O*(10^−6^) respectively. To evaluate the inertia of particle near a wall, we compare the hydrodynamic force by particle-wall interaction (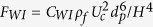, where *C*_*WI*_ is the coefficient varying from 0.2 to 0.8^41^,^42^) with the viscous drag on a Brownian particle of equal size (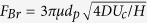), where diffusivity of a spherical Brownian particle is *D* = *k*_*B*_*T*/(3*π* *μd*_*p*_) and *k*_*B*_ is the Boltzmann constant). Given the flow conditions and characteristics of tracer particle, the *F*_*WI*_ is on the order of *O*(10^−26^) versus that of *F*_*Br*_ at *O*(10^−14^). It can be concluded that inertia of particle in current experiments is negligible.

#### Numerical removal of roughness interference from hologram

The interference patterns of the pillars (Fig. 1b) are clearly observed overwhelming those of tracer particles. To measure the position and motion of tracers accurately overcoming these interferences, they must be removed from the hologram. We applied a de-noising algorithm to estimate the background noise hologram containing only the interference of pillars and laser instability for each individual hologram^40^. Data analysis includes the following steps: (i) The original holograms were low-pass filtered first to remove the small scale particle interference patterns, while keeping the large scale background noise features like pillars; (ii) the filtered images were processed by a correlation algorithm to compute the similarity of each hologram with the rest of dataset, i.e. indicated by correlation coefficients ranging between 0 and 1; and (iii) a background hologram for each individual holographic recording containing only the interference of pillar were constructed based on ensemble averaging over a series of similar holograms among which the correlation coefficient of any two holograms exceeds 0.7. Note that although the low-pass filtered images were used in coefficient calculation, the actual background holograms are constructed from the original holograms. Figure 1c shows the portion of the same sample hologram (Fig. 1b) after background removal. The enhancement of tracer hologram is clearly evident, allowing us to measure the fluid motion very close to those pillars. The background hologram were reconstructed separately to extract the information on the position, shape and motion of pillars.

## Additional Information

**How to cite this article**: Evans, H. B. *et al.* Holographic microscopy and microfluidics platform for measuring wall stress and 3D flow over surfaces textured by micro-pillars. *Sci. Rep.* **6**, 28753; doi: 10.1038/srep28753 (2016).

## Figures and Tables

**Table 1:** Characteristics of microfluidics and pillars.

Dimension of microchannel			Characteristics of pillar			Surface property		
Height (H)	Width (W)	Length (L)	Diameter (*d*_*mp*_)	Height (*h*_*mp*_)	Roughness ( 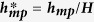 )	Pitch Δ*x*, Δ*y*	Top (Glass)	Bottom (PDMS)
1.42	10 *mm*	45	40 *µm*	40 *µm*	0.028	120 *µm*	Hydrophilic	Hydrophobic

The subscript, “*mp*”, refers to pillar.

**Table 2:** Five investigation cases characterized as flow rate (*Q*), centerline velocity (*U*_*c*_), streamwise wall shear (*du*/*dz*), corresponding recording rate and Re based on channel height, were conducted, totaling 11,300 holograms per case.

*Q *(*mL*/*min*)	*U*_*c*_* *(*mm*/*s*)	*du*/*dzs*^−1^	Frame Rate (FPS)	*Re*_*H*_* *(*U*_*c*_*H*/*ν*)
0.5	1.25	3.6	100	1.8
**1.1**	**2.4**	**7.2**	**200**	**3.4**
2.0	5.0	14.4	400	7.1
5.0	12.5	35.9	1000	17.8
10.0	25.0	71.8	2000	35.5
